# Questionnaire-based detection of immune-related adverse events in cancer patients treated with PD-1/PD-L1 immune checkpoint inhibitors

**DOI:** 10.1186/s12885-021-08006-0

**Published:** 2021-03-24

**Authors:** Luisa Maria Griewing, Claudia Schweizer, Philipp Schubert, Sandra Rutzner, Markus Eckstein, Benjamin Frey, Marlen Haderlein, Thomas Weissmann, Sabine Semrau, Antoniu-Oreste Gostian, Sarina K. Müller, Maximilian Traxdorf, Heinrich Iro, Jian-Guo Zhou, Udo S. Gaipl, Rainer Fietkau, Markus Hecht

**Affiliations:** 1 Department of Radiation Oncology, Universitätsklinikum Erlangen, Friedrich-Alexander-Universität Erlangen-Nürnberg, Universitätsstraße 27, 91054 Erlangen, Germany; 2 Comprehensive Cancer Center Erlangen-EMN, Erlangen, Germany; 3 Institute of Pathology, Universitätsklinikum Erlangen, Friedrich-Alexander-Universität Erlangen-Nürnberg, Erlangen, Germany; 4 Department of Otolaryngology - Head & Neck Surgery, Universitätsklinikum Erlangen, Friedrich-Alexander-Universität Erlangen-Nürnberg, Erlangen, Germany; 5 Department of Oncology, The Second Affiliated Hospital of Zunyi Medical University, Zunyi, China

**Keywords:** Immune checkpoint inhibitors, PD-1, Immune-related adverse events, Toxicity, Questionnaire, PD-L1, Side effects, Solid tumors, Patient-reported irAE

## Abstract

**Background:**

Immune checkpoint inhibitors (ICI) have become standard treatment in different tumor entities. However, safe treatment with ICI targeting the PD-1/PD-L1 axis requires early detection of immune-related adverse events (irAE). There exist different questionnaires of drug manufacturers for the detection of irAE that have not been validated so far.

**Methods:**

The prospective non-interventional ST-ICI trial studied treatment with PD-1/PD-L1 ICI alone or combined with radiotherapy. In the current analysis, the detection rate of self-reported irAE with a patient questionnaire containing 41 different questions was compared to clinician-reported irAE.

**Results:**

Between April 2017 and August 2019, a total of 104 patients were prospectively enrolled. NSCLC (44%) and HNSCC (42%) were the most frequent tumor entities. A total of 784 questionnaires were collected. A total of 29 irAE were reported by clinicians. The most frequent irAE was hypothyroidism (9%), followed by skin reactions (5%), hepatitis (4%), diarrhea (3%), and pneumonitis (3%). Questions that became significantly more often positive at time points of clinician-reported irAE were “weight change”, “difficulty to grip things”, “bloody or mucous stool” and “insomnia”. Self-reported organ-specific questions detected at least 50% of clinician-reported irAE of gastrointestinal, lung, endocrine, and skin irAE. It was not possible to detect hepatic irAE with the questionnaire.

**Conclusion:**

Questionnaires can help to detect gastrointestinal, lung, endocrine, or skin irAE, but not hepatic irAE. Questions on “weight change” and “insomnia” may help to increase the detection rate of irAE, besides organ-specific questions. These results are a valuable contribution to the future development of a specific and practicable questionnaire for early self-reported detection of irAE during ICI therapy in cancer patients.

**Trial registration:**

ClinicalTrials.gov, NCT03453892. Registered on 05 March 2018.

**Supplementary Information:**

The online version contains supplementary material available at 10.1186/s12885-021-08006-0.

## Background

Over the last decade, treatment with immune checkpoint inhibitors (ICI) against programmed cell death protein 1 (PD-1) and one of its ligands PD-L1 have become one of the most promising approaches in the field of cancer therapy. Consequently, their application in oncologic treatment is continuously increasing [1, 2]. However, despite the advantages of ICI therapy, a valid predictive biomarker that accurately identifies patients who will benefit from ICI treatment has yet to be developed. The expression of PD-L1 on tumor cells and/or immune cells has been identified as one of such immune biomarkers. Nevertheless, the expression of PD-L1 is not stable as it is e.g. up-regulated by radiochemotherapy [3–6]. Also the tumor mutational burden or the intratumoral CD8 cell density may serve as a predictive marker [7, 8]. The great success of ICI is shadowed by the induction severe immune-related adverse events in some patients. The gastrointestinal, pulmonary, dermatologic, hepatic, and endocrine systems are thereby frequently affected [1, 9, 10]. The ASCO Clinical Practice Guideline Summary presents a system-based toxicity diagnosis and management guideline, which recommends treatments depending on the affected system [11, 12]. Several studies have shown that the occurrence of immune-related adverse events (irAE) might have a positive influence on tumor treatment response and the survival rate [13–17]. As a result, the onset of irAE represents a potentially beneficial clinical marker for ICI efficacy and should be further evaluated also in this respect. However, the occurrence and frequency of reported adverse events (AE) differ between patient self-reports and diagnoses of the managing clinicians. In this regard, patients tend to report AE more often and earlier. Consequently, it seems appropriate to increase patient self-reports using appropriate questionnaires for timely detection and subsequent therapy of AE [18, 19]. Pharmacokinetics of ICI allow dose intervals of up to 6 weeks [10, 20]. These long dose intervals bear the risk that irAE may be detected late, which impairs patients’ safety. Patient self-reports on irAE may help to ensure an early detection of irAE despite these long dose intervals.

There exist different questionnaires of drug manufacturers for the detection of irAE that have not been validated so far. Adapted from these questionnaires an own questionnaire was developed for patients’ self-reported detection of irAE. The aim of the current analysis is to compare the patients’ self-reported irAE to clinician-reported irAE.

## Methods

### Patients

Patients with non-melanoma solid tumors and the indication for ICI treatment with either a PD-1 or PD-L1 inhibitor were eligible for the trial. Concomitant radiotherapy was not obligatory. To represent an unselected cohort, there was no restriction concerning baseline Eastern Cooperative Oncology Group (ECOG) performance status, pre-existing diseases, tumor entity, or blood parameters.

### Trial design and treatments

ST-ICI is a prospective non-interventional, non-randomized trial in tumor patients treated with ICI. Patients receive either immunotherapy in combination with radiotherapy or immunotherapy alone. A secondary endpoint of the current interim analysis is the detection rate of irAE using a newly developed questionnaire. All treatment decisions are made by treating physicians based on clinical standards and national guidelines. Any EMA-approved PD-1 or PD-L1 inhibitor treatment was allowed within the trial. Dose and the treatment indication of the ICI were according to the EMA marketing authorization. Radiotherapy was delivered either as stereotactic radiosurgery or fractionated radiotherapy.

### Endpoints and assessments

**Fig. 1:**
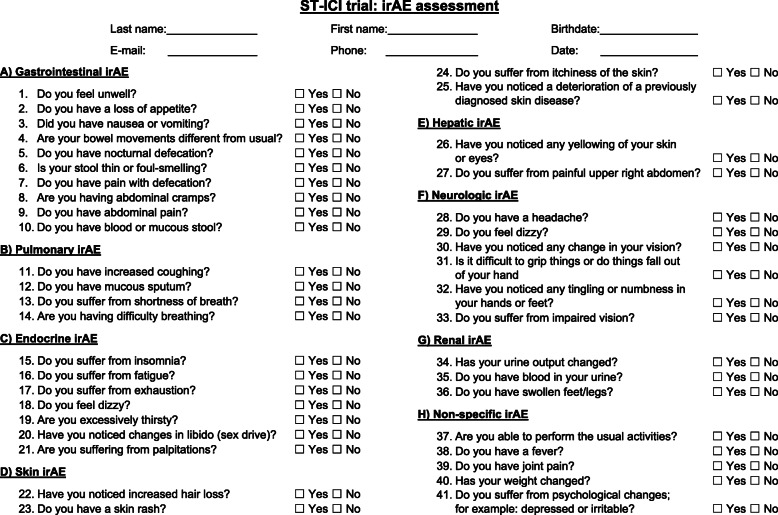
ST-ICI trial: irAE assessment. Assessment of irAE with easily routinely usable binary response questions (yes/no) focusing on the most affected organ systems

The current analyses focus on a secondary endpoint of the ST-ICI trial. In this explorative interim analysis the detection of different irAE with a newly developed 41 item irAE questionnaire is studied (Fig. 1). The questionnaire consists of eight multi-item symptom scales (gastrointestinal, pulmonary, endocrine, skin, hepatic, neurologic, renal, and non-specific). All symptom scales contain several binary response questions (“yes” or “no”) to identify specific irAE. Patients were asked to complete the questionnaire for irAE before each administration of the PD-1 or PD-L1 inhibitor, i.e. typically every second or third week beginning with the first administration. Patients had to complete the questionnaire in a written form without support from the medical personal and before the contact to the clinician. The clinician-reported irAE were assessed routinely before every administration of the drug and in case of new symptoms. The questionnaire was not visible to the clinician. The clinician-reported irAE were classified according to the Common Terminology Criteria of Adverse Events (CTCAE), version 5.0.

The primary objective of the presented work was to investigate whether irAE can be detected by organ-specific questions (e.g. diarrhea by gastrointestinal questions). Furthermore, questions concerning any irAE have been analyzed independently from their specific irAE, to identify general screening-questions for any irAE. This should help to identify appropriate questions for the development of a specific questionnaire on irAE, which can enter a future questionnaire validation process.

### Trial oversight

The registration of the ST-ICI trial is allocated in Clinical Trails.gov (identifier: NCT03453892). The leading institutional review board at the Friedrich-Alexander-Universität Erlangen-Nürnberg approved the study (number: 2_17 B). The written informed consent of all patients has been obtained. The study had no external funding. All methods were performed in accordance with the relevant guidelines and the Declaration of Helsinki.

### Statistical analysis

Due to the very low prevalence of irAE compared to the number of questionnaires the frequency of positively answered questions at time points of clinician reported irAE will be presented descriptively. Due to this imbalance of the high number of questionnaires and low number of irAE classical parameters as sensitivity, specificity, positive and negative predictive values were waived. It is the aim of this explorative analysis of this prospective trial to identify appropriate questions for the development of a specific questionnaire that can enter a classical validation process. IBM SPSS Statistics version 24 was used for performing all statistical tests. Fisher’s exact test was used to study differences in the frequency of positively answered questions in the subgroups with and without irAE. *P*-values below 0.05 level are expected to be statistically significant. The statistical analysis was done for all irAE, the types of irAE were not analyzed separately.

## Results

### Patients and treatment

The ST-ICI trial registered 104 patients between April 2017 and August 2019. Baseline characteristics of the patients are given in Table 1. The median age was 66 years and 73% were male. Thirty-six patients (35%) had PD-L1 negative tumors (PD-L1 < 1%). All patients received immunotherapy with a PD-1 or PD-L1 inhibitor. Out of these, 50 patients (48%) received additional radiotherapy within 30 days before or after an application of immunotherapy. Detailed information on radiotherapy has been previously reported [21]. Non-small cell lung cancer (NSCLC, 44%) and head and neck squamous cell cancer (HNSCC, 42%) were the most frequent tumor entities. The remaining tumor entities in the cohort represented bladder cancer (5%), oesophageal cancer (4%), and other tumor entities (5%). The most frequent comorbidities were arterial hypertonia (33%) followed by other cardiovascular comorbidities (29%), diabetes mellitus (19%), and COPD (17%). Two patients with autoimmune diseases were included (2%). The majority of patients received the ICI nivolumab (64%) followed by pembrolizumab (23%) and durvalumab (9%). Immunotherapy was given in a palliative setting in 92 patients (88%) and an adjuvant setting in 12 patients (12%). The patients with palliative treatment included 15 patients (14%) with local tumor recurrence only and 77 patients (74%) with distant metastases, respectively. The median follow-up time was 8.3 months, whereas questionnaire-based irAE assessment was discontinued at the end of immunotherapy.

**Table 1:** Patient characteristics of the ST-ICI cohort

Patient characteristics	All patients *n* = 104 (%)
**Sex**	
Male	76 (73)
Female	28 (27)
**Median age ± SD** (years)	66 ± 10.2
**Treatment arm**	
IT-RT	50 (48)
IT-only	54 (52)
**Location of radiotherapy**^a^ (*n* = 50)	
Lung	17 (34)
CNS	10 (20)
Bone	5 (10)
Other	18 (36)
**PD-L1 tumor cells**	
< 1%	36 (35)
1–49%	33 (32)
≥ 50%	32 (31)
Unknown	3 (3)
**Brain metastases**	24 (23)
**Tumor entity**	
NSCLC	46 (44)
HNSCC	44 (42)
Bladder cancer	5 (5)
Oesophageal cancer	4 (4)
Other	5 (5)
**Number of previous treatments**	
0–1	61 (59)
≥ 2	43 (41)
**Disease stage**	
**Adjuvant setting**	12 (12)
**Palliative setting**	92 (88)
local tumor recurrence	15 (14)
distant metastases	77 (74)
**Drug**	
Nivolumab	66 (64)
Pembrolizumab	24 (23)
Durvalumab	9 (9)
Other	5 (5)
**Concomitant chemotherapy**	6 (6)
**Comorbidity**^b^ (*n* = 199)	
Arterial hypertonia	34 (33)
Cardiovascular comorbidity	30 (29)
Diabetes	20 (19)
COPD	18 (17)
Autoimmune disease	2 (2)
Other	87 (84)
None	8 (8)

^a^Only radiotherapy within 30 days before or after first administration of ICI

^**b**^Multiple co-morbidities per patient possible

*SD* Standard deviation, *IT* Immunotherapy, *RT* Radiotherapy, *CNS* Central nervous system, *PD-L1* Programmed death ligand, *HNSCC* Head and neck squamous cell carcinoma, *NSCLC* Non small cell lung carcinoma

### Incidence and grades of irAE

Twenty-nine out of one hundred four surveyed patients developed any irAE (Table 2). The median time from first drug administration to the endpoint clinician-reported irAE was 2.3 months. Hypothyroidism was the most frequent one (9%), followed by skin reactions (5%), hepatitis (4%), diarrhea (3%), pneumonitis (3%), and other irAE (5%). According to CTCAE 25 patients (24%) were classified to grade 1–2 reactions while four patients (4%) developed grade 3 irAE. No grade 4 and 5 events were observed. All cases of hypothyroidism and hepatitis were confirmed by laboratory tests, skin reactions were evaluated clinically, all cases of pneumonitis were confirmed by CT scan. Colonoscopy in case of diarrhoea was not mandatory. In five patients irAE led to immunotherapy treatment interruption and in six patients to permanent discontinuation. In the two patients with pre-existing autoimmune diseases, one patient with psoriasis experienced a disease flare and one patient with Hashimoto’s thyroiditis in the hypothyroid stage experienced no irAE. As mentioned above, some patients received radiotherapy in addition to immunotherapy. In order to clarify that irAE in these patients are no local radiotherapy effects, these cases are summarized in supplementary Table 1.

**Table 2:** Immune-related adverse events

irAE^a^	Any Grade	Grade 1–2	Grade 3
	*n =* 104 (%)	*n =* 104 (%)	*n =* 104 (%)
Any irAE	29 (28)	25 (24)	4 (4)
Hypothyroidism	9 (9)	9 (9)	0 (0)
Skin reaction	5 (5)	5 (5)	0 (0)
Hepatitis	4 (4)	1 (1)	3 (3)
Diarrhea	3 (3)	3 (3)	0 (0)
Pneumonitis	3 (3)	3 (3)	0 (0)
Other	5 (5)	4 (4)	1 (1)

^a^*irAE* Immune-related adverse event. No grade 4 and 5 events were observed

### Patient irAE screening questionnaire

**Fig. 2:**
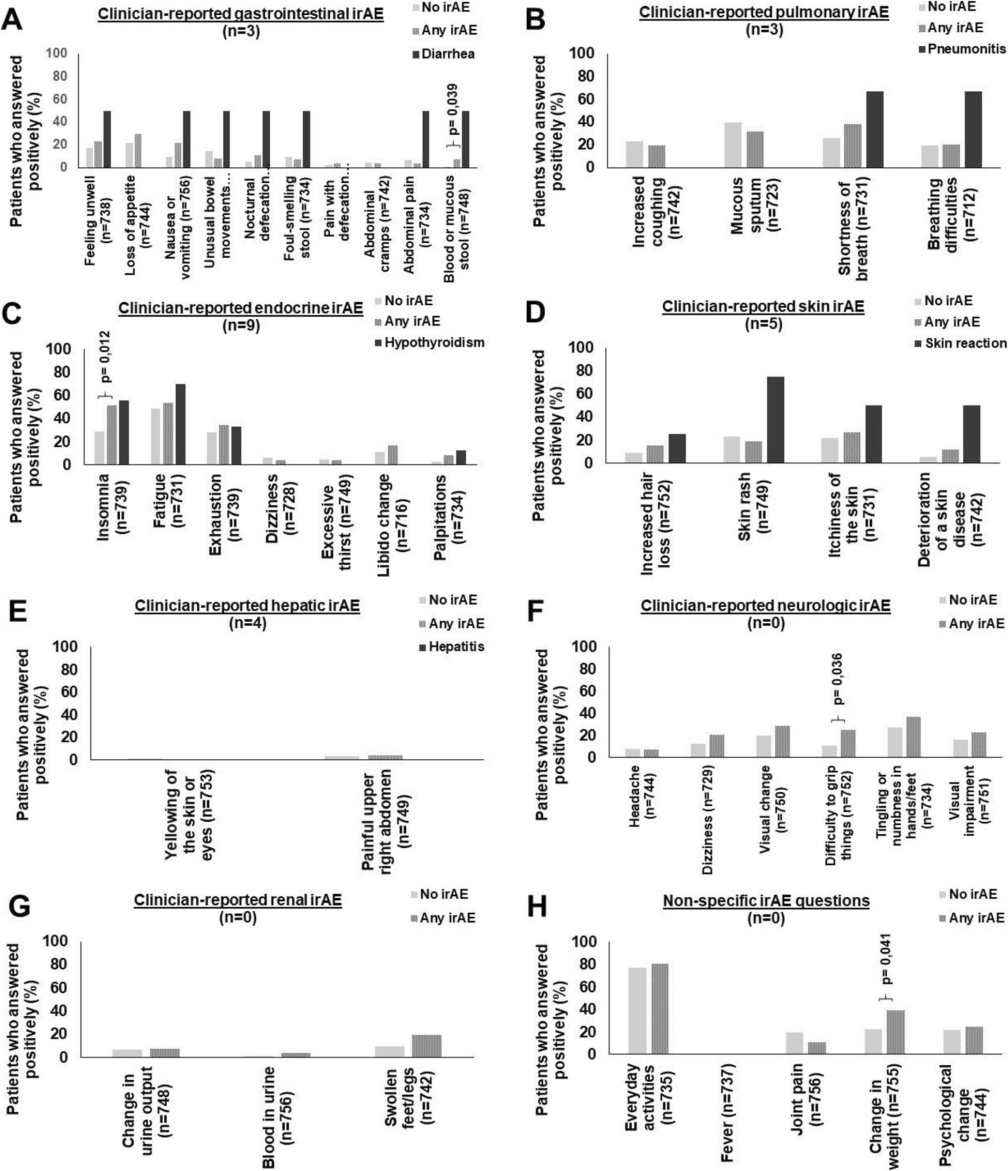
Frequency of positive answers of the ST-ICI cohort depending on clinician-reported irAE. **a** Gastrointestinal irAE. **b** Pulmonary irAE. **c** Endocrine irAE. **d** Skin irAE. **e** Hepatic irAE. **f** Neurologic irAE. **g** Renal irAE. **h** Non-specific irAE

A total of 784 questionnaires were collected. Out of the 104 surveyed patients, 96 patients (92%) completed at least one questionnaire. The median number of completed questionnaires per patient was 6 (range 0–40). Figure 2a-h depicts the percentage for each question that was positively answered separately for patients with no current clinician-reported irAE, for patients with current clinician-reported irAE, and for those with current clinician-reported irAE related to the organ-specific panel.

Clinician-reported gastrointestinal irAE were detected by different questions at a rate of ≥50% as “feeling unwell”, “nausea or vomiting”, “differing bowel movements”, “nocturnal defecation”, “thin or foul-smelling stool”, “abdominal pain” and “bloody or mucous stool” (Fig. 2a). Questions on “loss of appetite”, “painful defecation” or “abdominal cramps” did not correlate with gastrointestinal irAE.

Clinician-reported pulmonary irAE were detected at a rate of ≥50% by the questions “shortness of breath” and “difficulty in breathing” (Fig. 2b). Patients with pulmonary irAE, i.e. pneumonitis, did not report suffering from “increased coughing” and “mucous sputum”.

Clinician-reported endocrine irAE were identified in at least ≥50% of cases by the question on “insomnia”. The question on “fatigue” increased in endocrine irAE to 70%, whereas it was also answered positively in 49% of patients without irAE (Fig. 2c). Questions on “exhaustion”, “dizziness”, “excessive thirst”, “libido changes” or “palpitations” did not identify endocrine irAE.

Clinician-reported skin irAE were detected at a rate of ≥50% by the questions “skin rash”, “itchiness” and “deterioration of skin diseases”, whereas the question “hair loss” was not increased in skin irAE (Fig. 2d). Hepatic irAE were not detected, neither by the question “yellowing of skin or eyes” nor the question on “painful upper right abdomen” (Fig. 2e). The questionnaire also contained a neurologic and renal question panel, whereas no neurologic irAE (Fig. 2f) and no renal irAE (Fig. 2g) appeared. Several non-specific questions were asked for the general detection of any irAE or rare irAE (Fig. 2h).

Besides the aim to detect irAE organ-specific with this questionnaire, also general questions for the detection of any irAE were searched. Generally, the positive rate of several questions increased in patients with clinician-reported irAE, especially in the gastrointestinal, endocrine, and neurologic question panel. Patients who developed any clinician-reported irAE complained more often about “weight change” (*p* = 0.041), “difficulty to grip things” (*p* = 0.036), bloody or mucous stool” (*p* = 0.039), and “insomnia” (*p* = 0.012) in the self-reporting questionnaire (Fig. 2).

## Discussion

ICI are nowadays frequently used in the treatment of cancer and are increasingly integrated in multimodal approaches with chemotherapy or radiotherapy in current clinical trials [22]. However, ICI represent a rather novel therapy that goes along with a new type of potentially life-threatening irAE. As described, they may affect any organ system [1, 23]. Consequently, accompanying irAE should be detected and treated early. In this regard, questionnaires are a potentially easy-to-handle and beneficial tool for the timely detection and sufficient treatment of irAE [10, 20].

Various clinical trials identified differences between severity and frequency when AE are reported and measured by patients themselves or by supervising clinicians. Frequently, patients report their symptoms earlier and more often. Their reporting is better associated with the daily health status, whereas the clinical CTCAE assessment predicts unfavorable clinical outcomes [18, 19, 24]. This led to the development of the PRO CTCAE to capture symptomatic adverse events by patient self-report in cancer clinical trials [25, 26]. The PRO-CTCAE is very comprehensive including 124 items representing 78 symptomatic toxicities.

The presented work used our newly designed binary response questionnaire focusing on the most affected organ systems by ICI, i.e. gastrointestinal, lung, endocrine, skin, liver, neurologic, and renal. The questions are based on questionnaires provided by the manufacturers of ICI. The final questionnaire contains 41 binary questions.

Dermal side effects as rash, itchiness, and vitiligo are frequently mentioned during PD-1/PD-L1 inhibitor therapy [23, 27, 28]. In the ST-ICI study we observed patients noticing skin changes (especially skin rash) by themselves, which they documented in the questionnaires. The most common gastrointestinal complications are diarrhea and colitis. An increasing number of positive answers to the different questions confirmed complications as diarrhea and colitis. It can be assumed that gastrointestinal irAE can be detected easily with questionnaires as well. Endocrine irAE include thyroid dysfunction, hypophysitis, diabetes mellitus, and primary adrenal insufficiency. Concerning thyroid dysfunction, hypothyroidism is the most common one showing unspecific symptoms as fatigue [29]. However, fatigue is a very frequent and non-specific symptom of cancer patients but did not prove as an appropriate indicator of endocrine irAE. Interestingly, patients with endocrine irAE frequently mentioned the complaint insomnia. Hepatitis is the most frequent hepatic irAE [23, 30]. Here the questionnaire could not detect any difference from the baseline, which is in line with our expectations as hepatitis can be detected early by elevation of transaminases and is clinically often asymptomatic or associated with non-specific symptoms such as weakness, fatigue, nausea, or vomiting [31]. Pneumonitis remains a rare but concerning complication of ICI therapy. It is often detected with dyspnea or other pulmonary symptoms [32, 33]. Patients who developed pneumonitis in our study reported shortness of breath and difficulty in breathing. Thus, pneumonitis can also be detected by questionnaires. Neurologic and renal irAE are rare, but severe complications of immunotherapy. Most of the renal irAE are presented as acute interstitial nephritis [34, 35]. In the ST-ICI study no patient developed neurological and renal irAE. Thus, the questions concerning neurological and renal irAE could only be examined for any other side effects. In total, the data from the questionnaire of the ST-ICI trial shows a relation between the patient-reported and clinician-reported irAE. Several questions indicate a specific irAE.

Besides the detection of an organ-specific irAE, general screening questions for irAE would be helpful to monitor patients with long dose intervals of ICI. Within the ST-ICI questionnaire, patients with irAE answered the questions on “weight change”, “difficulty to grip things”, “bloody or mucous stool” and “insomnia” significantly more often positive. Other than “bloody or mucopurulent stool” associated with colitis, it is not clear why the other responses became positive in the case of irAE. One could speculate that transient short-term hyperthyroidism could cause the insomnia. Furthermore, hyper- or hypothyroidism could be causative for the weight changes. These questions might serve as screening questions for irAE in future questionnaires.

Based on our results, future questionnaires can be reduced to one question per organ system as the inclusion of multiple questions per organ system did not increase the detection rate. In addition non-specific questions as “weight change” or “insomnia” might increase the detection rate of irAE.

A potential bias of the ST-ICI trial may be that patients filled in the questionnaire before their contact with the physician. This may have encouraged them to report their symptoms to the physician and increased the correlation between patient- and clinician-reported irAE. Clinician-reported irAE were diagnosed according to clinical standards, there was no standardized diagnostic workup. A further limitation of the ST-ICI cohort is that the used questionnaire was adapted from questionnaires of drug manufacturers and has not been validated before. However, there exist no validated questionnaires on self-reported irAE detection so far. Thus, the presented analysis is a first step to identify appropriate questions for the development of a specific irAE questionnaire that can enter a validation process. Due to the high number of collected questionnaires and low number of irAE the results are presented only descriptively. In the current trial the calculation of sensitivity, specificity, and positive/negative predictive value was not the aim of the trial and is not possible within this study design. The number of included patients and clinician-reported irAE is too low for such analyses. Altogether 29 irAEs were reported, including three cases of diarrhoea. This underrepresentation of the irAE diarrhoea might also be a consequence of the low patient number. The mixed patient cohort including several tumor entities might be rated as a further limitation. However, the development of irAE does not depend on the treated tumor entity. The inclusion of different tumor entities is probably a strength as in a patient cohort with a single tumor entity, special organ-specific symptoms might be overrepresented (e.g. breathing problems in NSCLC). The major strength of the ST-ICI trial is its prospective design. Furthermore, the collection of a high number of questionnaires (*n* = 784) and close monitoring of patients is in favor of the presented results. The extremely high rate of completed questionnaires (92% of included patients) is unique. This also proves the suitability of this questionnaire for daily routine use. As mentioned above, the identification of irAE is essential for the patients’ safety. There exists a mild correlation between a reduction of quality of life during radiochemotherapy and prolonged survival, which is probably a marker for treatment intensity [36]. However, in case of the occurrence of irAE, ICI treatment frequently has to be stopped and irAE management has to be started. In contrast to past assumptions, treatment interruptions and glucocorticoid use for irAE management are no obstacle for the treatment success. Recent analyses of our ST-ICI trial and other trials indicate that patients with irAE have a superior prognosis compared to patients without irAE [13–17].

## Conclusions

Questionnaires can help to detect gastrointestinal, lung, endocrine, or skin irAE, but not hepatic irAE. Questions on “weight change” and “insomnia” may help to increase the detection rate of irAE, besides organ-specific questions. These results are a valuable contribution to the future development of a specific and practicable questionnaire for early self-reported detection of irAE during ICI therapy in cancer patients.

## Supplementary Information

**Additional file 1: Supplementary Table 1.** Location of radiotherapy in patients with irAE.

## Data Availability

The data that support the findings of this study are available from the corresponding author upon reasonable request.

## Abbreviations

ASCO: *American Society of Clinical Oncology*
CTCAE: Common Terminology Criteria of Adverse Events
ECOG: Eastern Cooperative Oncology Group
EMA: European Medicines Agency
HNSCC: Head and neck squamous cell cancer
ICI: Immune checkpoint inhibitors
irAE: Immune-related adverse events
NSCLC: Non-small cell lung cancer
PD-1: Programmed cell death 1 protein
PD-L1: Programmed cell death ligand 1
PRO: Patient reported outcome
