# Supplementary material for: Questionnaire-based detection of immune-related adverse events in cancer patients treated with PD-1/PD-L1 immune checkpoint inhibitors
Source: BMC Cancer. 2021 Mar 24;21:314. doi: 10.1186/s12885-021-08006-0 (PMC7992796; doi:10.1186/s12885-021-08006-0)
Supplement: Supplementary file 1 — Additional file 1: Supplementary Table 1. Location of radiotherapy in patients with irAE. [file 12885_2021_8006_MOESM1_ESM.docx]

# Questionnaire-based detection of immune-related adverse events in cancer patients treated with PD-1/PD-L1 immune checkpoint inhibitors

Luisa Maria Griewing^1,4^, Claudia Schweizer^1,4^, Philipp Schubert^1,4^, Sandra Rutzner^1,4^, Markus Eckstein^2,4^, Benjamin Frey^1,4^, Marlen Haderlein^1,4^, Thomas Weissmann^1,4^, Sabine Semrau^1,4^, Antoniu-Oreste Gostian^3,4^, Sarina K. Müller^3,4^, Maximilian Traxdorf^3,4^, Heinrich Iro^3,4^, Jian-Guo Zhou^1,4,5^, Udo S. Gaipl^1,4^, Rainer Fietkau^1,4^, Markus Hecht^1,4^*

1 Department of Radiation Oncology, Universitätsklinikum Erlangen, Friedrich-Alexander-Universität Erlangen-Nürnberg, Erlangen, Germany

2 Institute of Pathology, Universitätsklinikum Erlangen, Friedrich-Alexander-Universität Erlangen-Nürnberg, Erlangen, Germany

3 Department of Otolaryngology - Head & Neck Surgery, Universitätsklinikum Erlangen, Friedrich-Alexander-Universität Erlangen-Nürnberg, Erlangen, Germany

4 Comprehensive Cancer Center Erlangen-EMN, Erlangen, Germany

5 Department of Oncology, The Second Affiliated Hospital of Zunyi Medical University, Zunyi, China

*Corresponding author: PD Dr. med. Markus Hecht, Department of Radiation Oncology, University Hospital Erlangen, Friedrich-Alexander-Universität Erlangen-Nürnberg, Universitätsstraße 27, D-91054 Erlangen, Germany, phone: +49 9131 8544247, fax: +49 9131 8539335, e-mail: markus.hecht@uk-erlangen.de

Supplementary Table 1: Location of radiotherapy in patients with irAE

| irAE description | Number of patients  with irAE | Location of radiotherapy |
| --- | --- | --- |
| Hypothyroidism | 6 | Lung, Bone, cervical lymph nodes, cardia lymph nodes |
| Skin reaction | 2 | Bone, thoracic lymph nodes |
| Hepatitis | 2 | Cervical lymph nodes, lung |
| Diarrhea | 2 | Cervical lymph nodes, kidney |
| Pneumonitis | 2 | Lung, brain |
| Other | 1 | lung |
